# Different laboratory populations similar bacterial profile? The case of Glossina palpalis gambiensis

**DOI:** 10.1186/s12866-018-1290-9

**Published:** 2018-11-23

**Authors:** Vangelis Doudoumis, Antonios Augustinos, Aggeliki Saridaki, Andrew Parker, Adly M M Abd-Alla, Kostas Bourtzis, George Tsiamis

**Affiliations:** 1 Department of Environmental and Natural Resources Management, University of Patras, 2 Seferi St, 30100 Agrinio, Greece; 2 Insect Pest Control Laboratory, Joint FAO/IAEA Division of Nuclear Techniques in Food and Agriculture, Vienna International Centre, P.O. Box 100, 1400 Vienna, Austria

**Keywords:** Tsetse, Amplicon sequencing, Bacterial profile, Symbiosis, Lab colonies

## Abstract

**Background:**

Microbiota plays an important role in the biology, ecology and evolution of insects including tsetse flies. The bacterial profile of 3 *Glossina palpalis gambiensis* laboratory colonies was examined using 16S *rRNA* gene amplicon sequencing to evaluate the dynamics of the bacterial diversity within and between each *G. p. gambiensis* colony.

**Results:**

The three *G. p. gambiensis* laboratory colonies displayed similar bacterial diversity indices and OTU distribution. Larval guts displayed a higher diversity when compared with the gastrointestinal tract of adults while no statistically significant differences were observed between testes and ovaries. *Wigglesworthia* and *Sodalis* were the most dominant taxa. In more detail, the gastrointestinal tract of adults was more enriched by *Wigglesworthia* while *Sodalis* were prominent in gonads. Interestingly, in larval guts a balanced co-existence between *Wigglesworthia* and *Sodalis* was observed. Sequences assigned to *Wolbachia*, *Propionibacterium*, and *Providencia* were also detected but to a much lesser degree. Clustering analysis indicated that the bacterial profile in *G. p. gambiensis* exhibits tissue tropism, hence distinguishing the gut bacterial profile from that present in reproductive organs.

**Conclusions:**

Our results indicated that age, gender and the origin of the laboratory colonies did not significantly influence the formation of the bacterial profile, once these populations were kept under the same rearing conditions. Within the laboratory populations a tissue tropism was observed between the gut and gonadal bacterial profile.

**Electronic supplementary material:**

The online version of this article (10.1186/s12866-018-1290-9) contains supplementary material, which is available to authorized users.

## Background

Tsetse flies (Diptera: Glossinidae) are the sole vectors of the African trypanosomes that cause sleeping sickness in humans (Human African Trypanosomiasis, HAT) and nagana in animals (Animal African Trypanosomiasis, AAT) [1, 2]. Recent conservative estimates suggest that there are more than 50 million people at risk including several thousand HAT cases every year [3]. In addition, the economic losses in agriculture due to AAT are estimated to be approximately US$ 5 billion per year [4]. Lack of effective vaccines, inefficient control with fly traps and concerns about protection of the environment and insecticide resistance urged scientists to develop alternative control methods [5]. The use of the sterile insect technique (SIT) in the frame of area-wide integrated pest management programs (AW-IPM) has proven to be successful in eradicating tsetse flies from Unguja Island (Zanzibar), United Republic of Tanzania [6].

In 2012, an SIT project was initiated in Senegal that aimed to eradicate a population of *Glossina palpalis gambiensis* (*Gpg*) from the Niayes area. The project was based on purchasing mass-reared sterile male tsetse pupae of a strain originating from Burkina Faso (BKF strain) from the rearing facilities at the Centre International de Recherche-Développement sur l’Élevage en zone Subhumide (CIRDES), Burkina Faso and the Slovak Academy of Sciences (SAS) in Slovakia. Both locations are rearing the same strain (BKF) that has been maintained for more than 35 years under artificial rearing conditions. Male pupae on the point of emergence were chilled to prevent emergence, irradiated and shipped twice weekly from CIRDES and SAS by air to Dakar, Senegal [7, 8].

Data from trial sterile male releases indicated high mortality and low recapture rate of the release sterile BKF males. In view of the higher mortality rates of the sterile males under field conditions in Senegal, an attempt was made at the Insect Pest Control Laboratory (IPCL), Seibersdorf, Austria, to establish a new colony from flies collected in the Niayes (SEN strain). Establishing and domesticating tsetse colonies is a slow and laborious process, so as soon as enough SEN material was available SEN males were outcrossed to BKF females to produce a line better adapted to rearing conditions. The introgressed flies were kept separate for four generations and then pooled to form the out-crossed colony (BKF-SEN strain). It was hoped that this BKF-SEN line would be better adapted to the harsh environmental conditions of Senegal. This colony was transferred to the SAS in Bratislava, Slovakia in mid-2012 for further colony build-up. In each generation, mating competitiveness tests were carried out in standard field cages where BKF-SEN males competed against BKF males for SEN females. The BKF-SEN strain progressively improved in mating participation with each generation. Once the BKF-SEN colony at the SAS reached a suitable size, shipments of sterile male pupae from BKF-SEN were sent to Senegal to assess its field performance. However, the performance of the BKF-SEN strain was worse than either the BKF or SEN strains in both the colony and under field conditions [9]. It was not clear that this poor performance could attributed among possible factors to the flies’ genetic background and / or the structure of the gut microbiota.

The bacterial communities in tsetse flies vary among species and seem to play a vital role in biology, fitness, immunology, reproduction and vectorial capacity [10, 11]. Tsetse flies have established sophisticated associations mainly with four bacterial symbionts. The first is the primary symbiont *Wigglesworthia*, a mutualist providing the host with important nutrients and vitamins as well as contributing to immunity [12–15]. The second is the facultative symbiont *Sodalis* which is present in most tsetse populations. This symbiont has been mainly localized in the midgut and associated with trypanosome infection [16]. The third is the reproductive parasite *Wolbachia*, which has been found in natural populations of tsetse flies with wide infection rates ranging from 0 to 100% [17, 18]. In addition, the *Wolbachia* strain present in *Glossina morsitans morsitans* (*Gmm*) can induce cytoplasmic incompatibility under laboratory conditions [18]. The fourth symbiont is *Spiroplasma*, representing a new class of tsetse symbiont found in *G. fuscipes fuscipes* (*Gff*), *G. tachinoides*, and *G. palpalis palpalis* (*Gpp*) [10]. *Spiroplasma* displays a tissue tropism with *Gff* testes exhibiting higher density than in ovaries. Furthermore, in *Gff* a putative mutualistic role for *Spiroplasma* was acknowledged [10].

Undoubtedly tsetse fly microbiota can be exploited since it is involved in several aspects of host biology and can be used for the development of innovative tools and strategies for vector and disease control [5, 19, 20]. So far, a limited number of culture-dependent and culture-independent studies have aimed to characterize the microbial composition in laboratory and natural populations of different species of tsetse flies [10, 11, 21]. *Enterobacter, Enterococcus*, and *Acinetobacter* species have been isolated from the guts of natural population of *Glossina palpalis palpalis* (*Gpp*) originating from Angola [22]. Geiger and colleagues also isolated a new bacterial species, *Serratia glossinae*, from the midgut of *Glossina palpalis gambiensis* (*Gpg)* insectary flies originating from individuals that had been field-collected in Burkina Faso [23]. Moreover, the gut bacterial composition of *Gpp* (*Acinetobacter* sp.*, Enterobacter* sp*., Enterococcus* sp.*, Lactococcus* sp.*, Providencia* sp.*, Staphylococcus* sp.*, Chryseobacterium* sp*.* and *Sphingobacterium* sp.), *Glossina pallicera* (*Enterobacter* sp.*, Acinetobacter* sp. and *Pseudomonas* sp.) and *Glossina nigrofusca* flies (*Enterobacter* sp.) from Cameroon have been investigated using culture-dependent techniques [24]. Utilizing both culture dependent and independent methods, Lindh & Lehane showed that Kenyan populations of *Glossina fuscipes fuscipes* (*Gff*) harbour an unexpected high diversity of bacteria including *Arthrobacter* sp*., Bacillus* sp*., Bacillaceae* sp*., Bacillales* sp*., Planococcaceae* sp*., Paenibacillus sp., Staphylococcus sp., Exiguobacterium sp., Burkholderia sp., Enterobacteriaceae, Pantoea* sp.*, Morganella* sp*., Providencia* sp*., Pseudomonas* sp*.* and *Serratia marcescens* [25]. On the other hand, a limited diversity of gut microbiota in several tsetse fly populations (*Gff*, *Gmm and G. pallidipes (Gpal*)) from Uganda was reported, based on multiple approaches, including deep sequencing of the V4 hypervariable region of the 16S *rRNA* gene, 16S *rRNA* gene clone libraries, and bacterium-specific quantitative PCR [21]. Recently, we showed for the first time that several wild individuals of *Gff*, *Gpp* and *G. tachinoides (Gt)*, which belong to the palpalis subgroup of the *Glossina* genus, were infected with *Spiroplasma* [10]. Additionally, in this study the microbial diversity (including members of the *Enterobacteriaceae* family, such as *Klebsiella, Erwinia, Trabulsiella, Pantoea*, and *Serratia*) of *G. medicorum* (*Gmed*), *G. morsitans submorsitans* (*Gms*), *Gpg* and *Gt* tsetse flies collected in Burkina Faso was identified by high throughput sequencing of the 16S *rRNA* gene [10]. In parallel, the bacterial composition of laboratory reared tsetse flies *Gff, Gmm*, and *Gpal* consists of additional bacterial species, such as members of *Flavobacterium, Propiniobacterium, Brevundimonas, Aeromonas, and Rhodospirillales* [10]. Also, sequences related to *Acinetobacter* and *Pantoea* were detected in *Gmm* and *Gpal*, while sequences similar to *Streptococcus* were identified in *Gmm*, and *Gff.* Finally, sequences related to *Shewanella* and *Pedobacter* were found only in *Gmm* [10].

In this study, we employed high throughput sequencing of the 16S *rRNA* gene to unravel the diversity of symbiotic bacteria in three laboratory populations of *Gpg* flies. Specifically, our research efforts focused on the investigation of microbiota in larval guts, and in guts and gonads of males and females collected at two age stages: (a) teneral and (b) 15-day-old insects from the three *Gpg* laboratory colonies. The aim was to assess the dynamics of bacterial diversity both within each *Gpg* laboratory colony and among them, in relation to the tissue distribution, insect age, gender and laboratory origin.

## Results

### *16S* rRNA gene amplicon sequencing of three *Gpg* laboratory colonies

#### Changes in alpha-diversity in relation to tissue, origin, age and gender

**Fig. 1:**
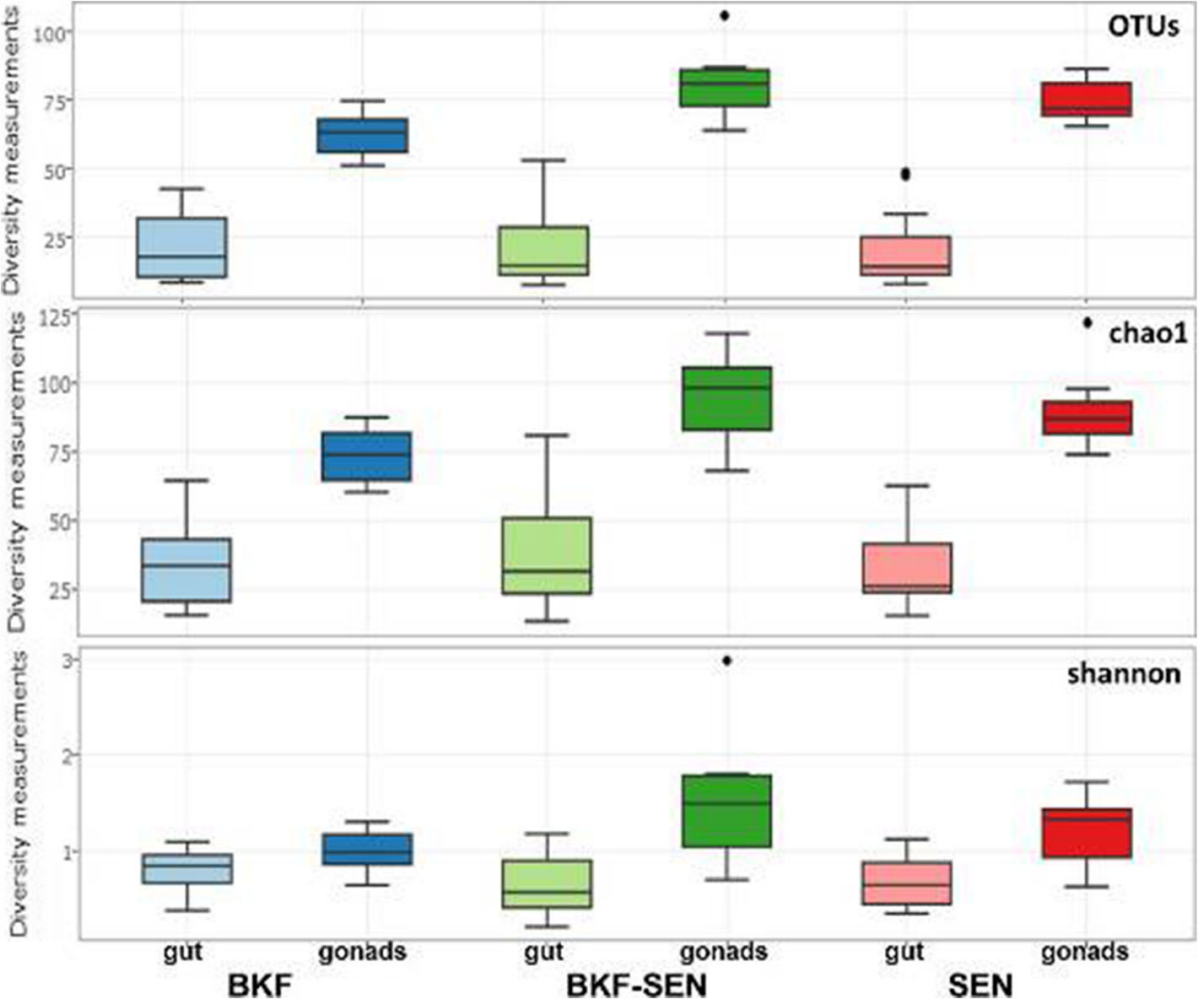
Species richness and diversity indices of the three examined *Gpg* laboratory colonies (BKF, BKF-SEN, and SEN) based on the number of OTUs, and the Chao1 and Shannon indices. Boxes denote the interquartile range, the line within the box is the median, and whiskers extend to the most extreme value within 1.5 *interquartile range from all samples (guts and gonads from teneral and 15-day old adults, as well as larval gut samples) examined for each colony. Outliers are indicated with dots

Bacterial community composition and diversity of the BKF, SEN and BKF-SEN *Gpg* laboratory colonies were investigated by 16S *rRNA* gene amplicon sequencing. In details, tissue samples (guts and gonads) from two developmental stages (teneral and 15-day old adults) originating from the three *Gpg* laboratory populations, as well as larval samples, were sequenced producing 3,469,061 reads after quality filtering. Overall, the three examined *Gpg* laboratory populations exhibited similar species richness and diversity indices based on number of operational taxonomic units (OTUs), Chao1 and Shannon indices (Fig. 1 and see Additional file 1).

**Fig. 2:**
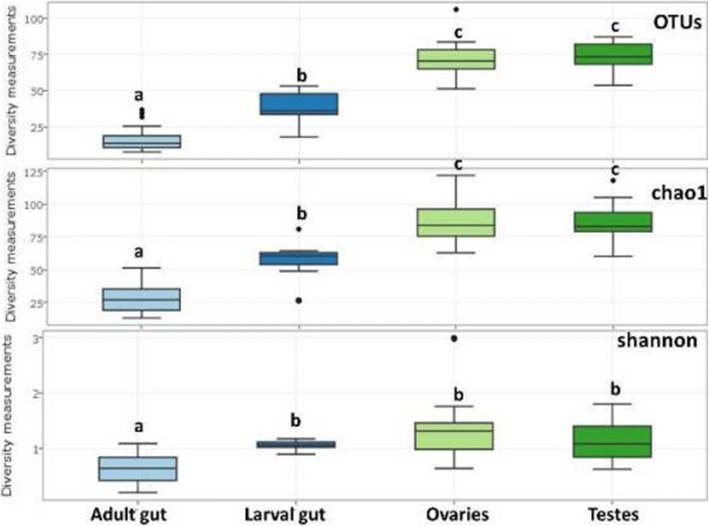
Alpha-diversity measurements of tissue samples from adult gut, larval gut and reproductive tissues represented as a pool of the three examined *Gpg* laboratory colonies based on the number of OTUs, and the Chao1 and Shannon indices. Boxes denote the interquartile range, the line within the box is the median, and whiskers extend to the most extreme value within 1.5 *interquartile range. Outliers are indicated with dots (*p* < 0.003; *t-test* was performed; significant differences are indicated by letters)

For all three analyzed *Gpg* laboratory colonies, the most bacterial species-rich samples were those associated with gonads while adult gut samples displayed a less bacterial species-rich profile based on the number of OTUs, and the Chao1 and Shannon indices (Fig. 2). In more details, testes exhibited statistically significant (t-test; *p* < 0.001) higher species-richness and diversity indexes when compared with adult guts (Fig. 2), regardless of the *Gpg* laboratory colony examined (data not shown). Similar statistically significant trend was observed among ovaries and adult guts, based on all indices (t-test; *p* < 0.003) (Fig. 2). Larval gut samples of all three *Gpg* lab colonies displayed statistically significant higher species richness and diversity indices (t-test; *p* < 0.001) when compared with adult guts (Fig. 2). Also, gonads displayed a statistically significant much bacterial species-richer profile than larval gut samples (t-test; *p* < 0.0002), based on the number of OTUs and the Chao1 indices (Fig. 2).

**Fig. 3:**
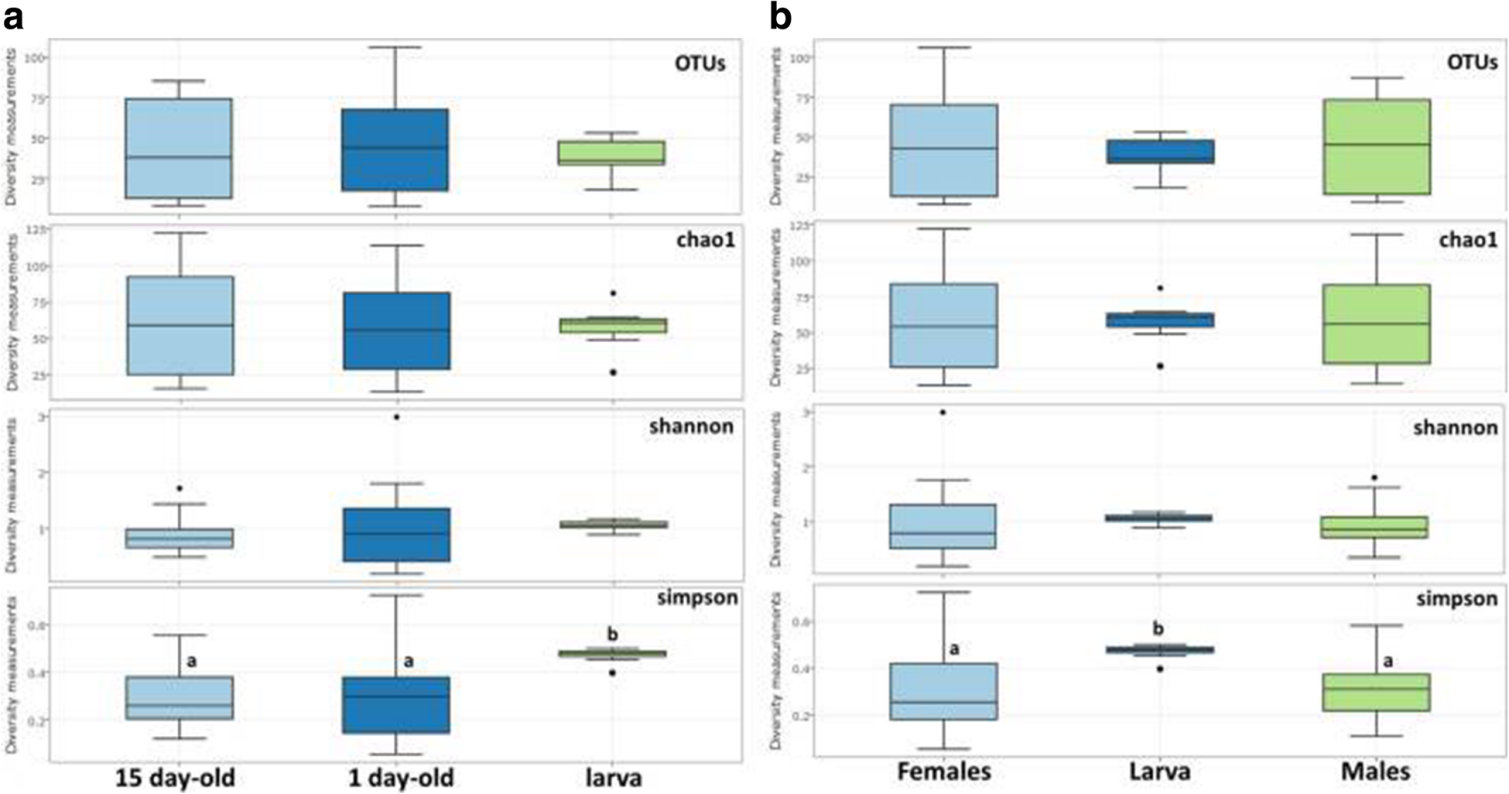
Alpha-diversity based of all *Gpg* samples examined classified on: (**a**) adult age and developmental stage (1 day-old, 15 day-old adults, and larva), (**b**) gender and developmental stage (Female, Male and larva). Boxes denote the interquartile range, the line within the box is the median, and whiskers extend to the most extreme value within 1.5 *interquartile range. Outliers are indicated with dots (*p* ≤ 0.0022; *t-test* was performed; significant differences are indicated by letters)

Age and gender do not significantly affect the species richness and diversity of the *Gpg* colony samples (Fig. 3). However, larval samples exhibited statistically significant (t-test; *p* < 0.0018) higher diversity Simpson index when compared with older (15-day old age and 1-day old age) samples, as well as with female and male samples (t-test; *p* < 0.0022) (Fig. 3a and b, respectively).

#### Dynamics of the bacterial diversity in relation to tissue, origin, age and gender

**Fig. 4:**
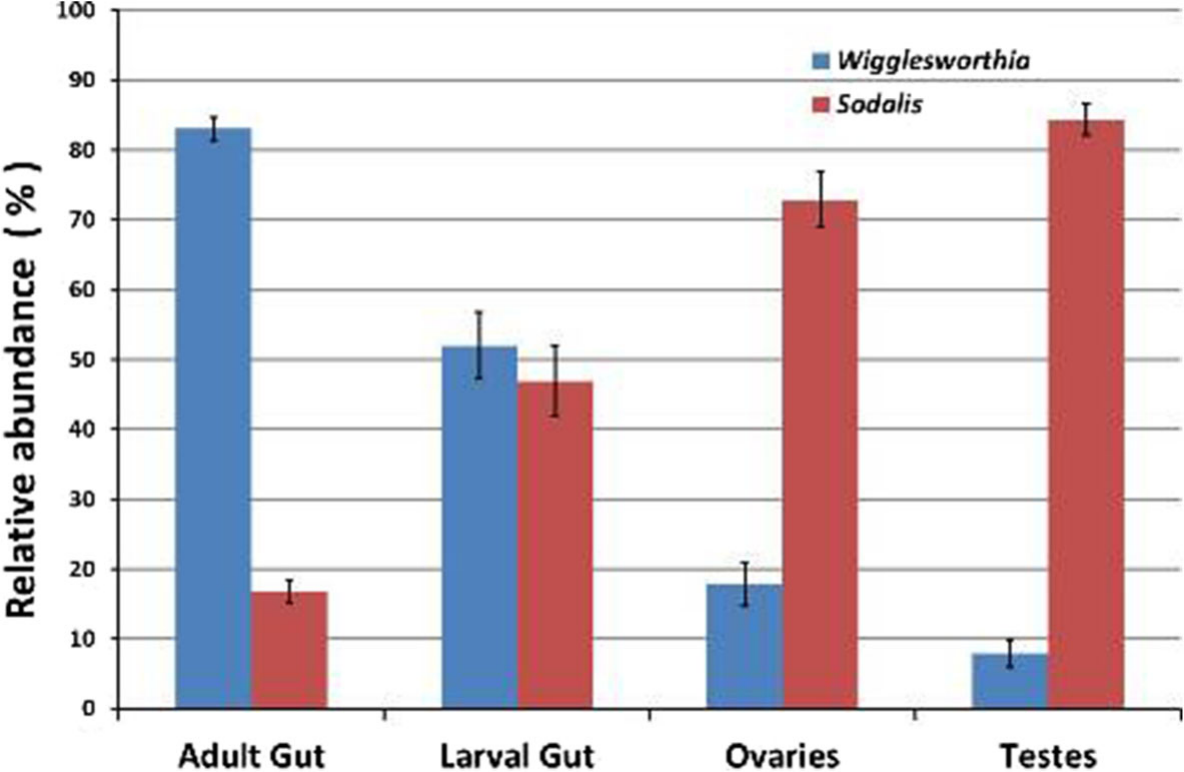
Relative abundance of *Wigglesworthia* and *Sodalis* (the most dominant taxa observed) among four tissue sample types (adult gut, larval gut, ovaries and testes) of all *Gpg* laboratory colonies examined

*Wigglesworthia* and *Sodalis* were the most dominant taxa in all analyzed samples. Relative abundance was influenced by tissue sample type with adult gut tissues dominated by *Wigglesworthia* while gonads were characterized by the dominance of *Sodalis*. In larval gut samples, a balanced co-existence between *Wigglesworthia* and *Sodalis* was observed (Fig. 4).

**Fig. 5:**
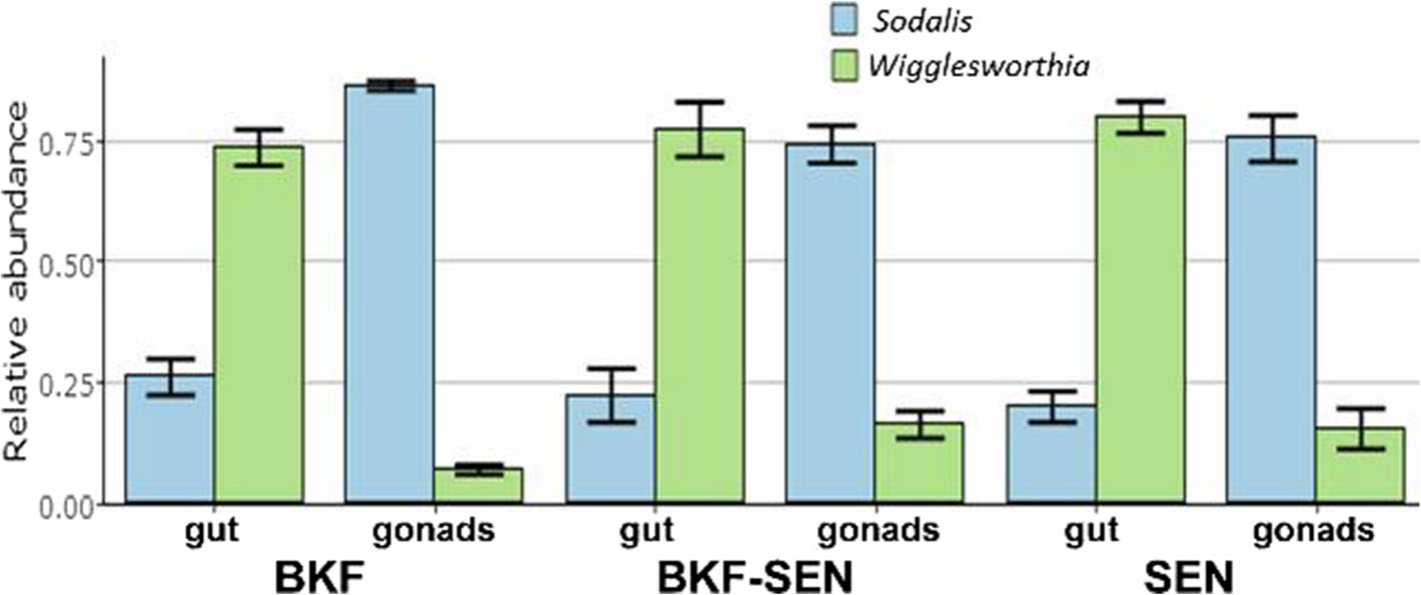
Relative abundance of *Wigglesworthia* and *Sodalis* (the most dominant taxa observed) in relation to the origin of the three *Gpg* laboratory colonies (BKF, BKF-SEN and SEN)

The primary nutritional endosymbiont of tsetse flies *Wigglesworthia glossinidia* was the most abundant taxon in all adult gut samples, and constituted between 72 and 97% of the total community in each adult gut sample. Conversely, in gonads tissue samples the facultative symbiont *Sodalis glossinidius* was the most dominant taxon, with its relative abundance ranging from 51 to 92% of the total community. In larval gut samples, the *Sodalis*:*Wigglesworthia* ratio was balanced for all three *Gpg* lab colonies (BKF 47%:53%, SEN 38%:60% and BKF-SEN 56%:43%), respectively (Fig. 5). In general, *Wigglesworthia* was the most dominant taxon in the laboratory colonies SEN and BKF-SEN, while *Sodalis* was dominating BKF (Fig. 5).

Age, gender and the origin of tsetse flies do not affect the relative abundance of the most dominant taxa in all analyzed samples (see Additional files 2, 3 and Fig. 5). However, in 15-day-old age tissue samples, the relative abundance of *Sodalis* is slightly higher than *Wigglesworthia*, while the opposite was noted in teneral (1 day-old age) and larval tissue samples (see Additional file 3).

**Fig. 6:**
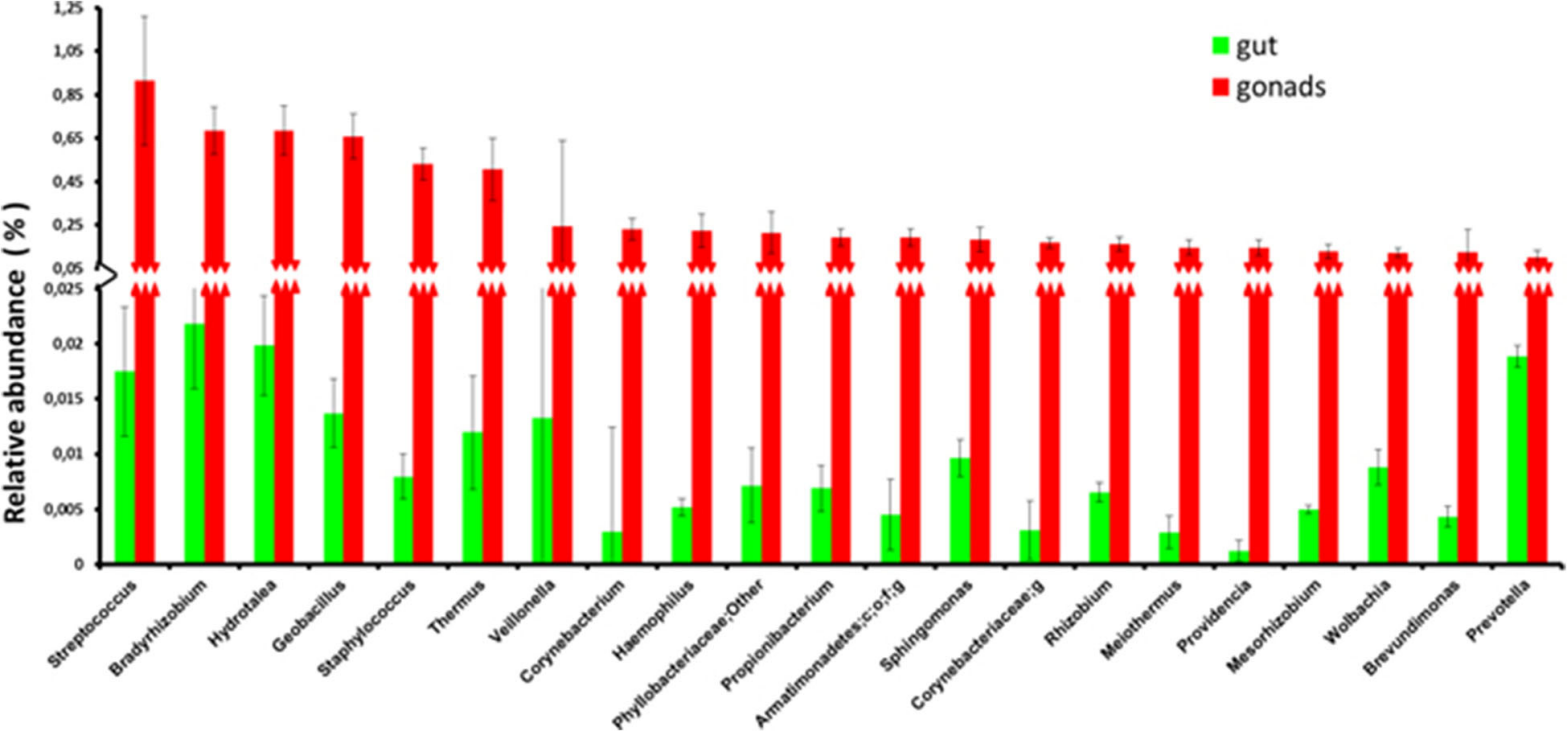
Relative abundance of taxa (> 0.05%) that were detected in *Gpg* tissue samples after exclusion of *Wigglesworthia* and *Sodalis*

In addition to *Wigglesworthia* (total mean abundance 48.4%) and *Sodalis* (total mean abundance 47.6%), several other taxa (exhibiting total mean abundance > 0.05%) were detected. These include multiple members of the *Gammaproteobacteria* (such as *Haemophilus* and *Providencia*), the *Bacilli* (such as *Streptococcus, Geobacillus.* and *Staphylococcus,)* the *Alphaproteobacteria* (such as *Bradyrhizobium, Phyllobacteriaceae, Sphingomonas, Rhizobium, Mesorhizobium*, *Wolbachia* with total mean abundance 0.06% and *Brevundimonas*), the *Sphingobacteria* (such as *Hydrotalea*), the *Actinobacteria* (such as *Corynebacterium* and *Propionibacterium*), the *Deinococci* (such as *Thermus* and *Meiothermus*), the *Clostridia* (such as *Veillonella*), the *Armatimonadetes* and the *Bacteroidia* (such as *Prevotella*) (Fig. 6).

**Fig. 7:**
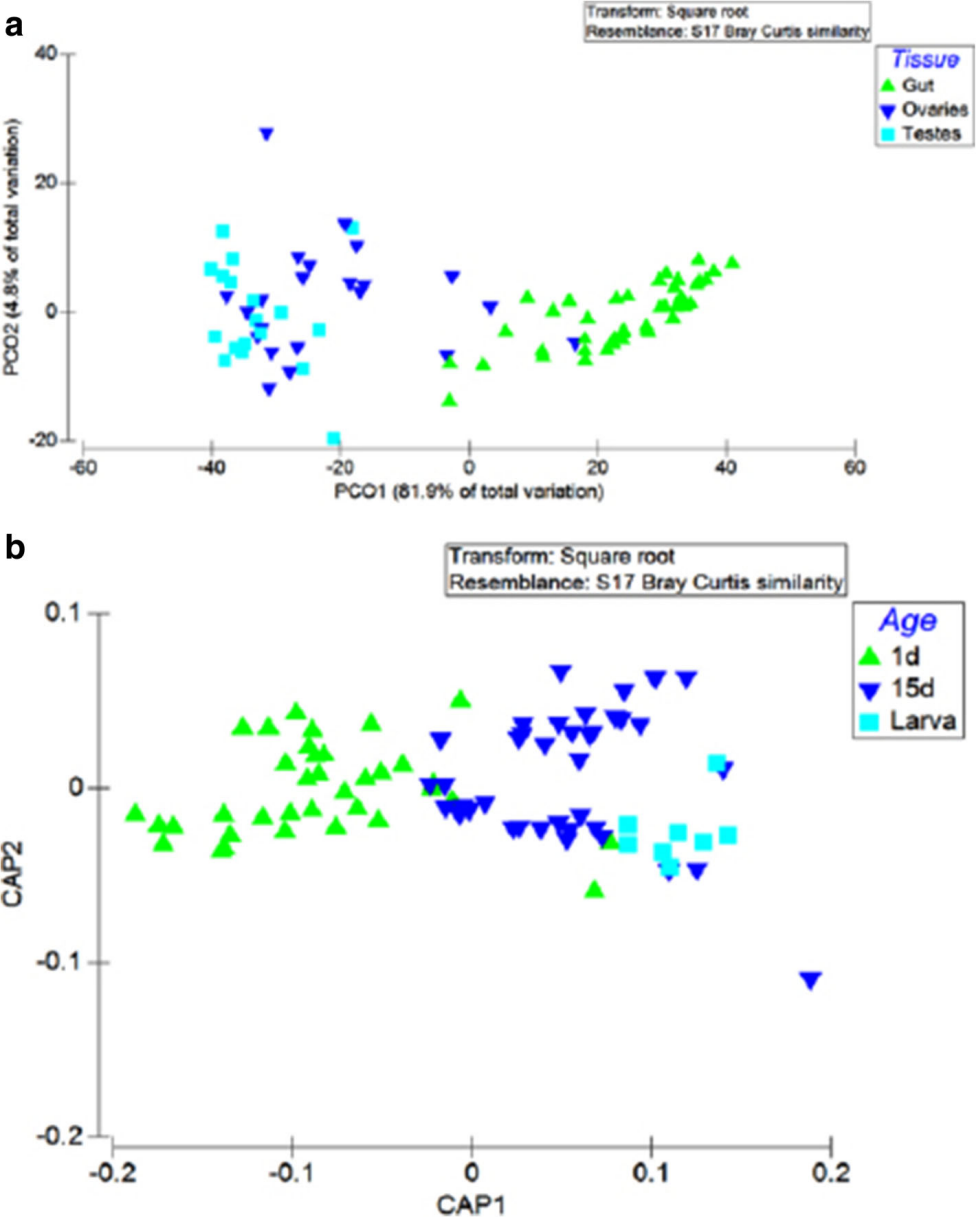
**a** Principal Coordinates Analysis (PCoA) of bacterial communities based on relative abundances of OTUs with originations from gut, ovaries, and testes of *Gpg* laboratory colonies. Variance explained by each PCoA axis is given in parentheses. **b** Canonical analysis of principal coordinates (CAP) ordinations of gut and bacterial communities based on relative abundances of OTUs from the three laboratory colonies. The constrained ordinations show maximized differences among the age of the adult insects and the developmental stage (trace_Q_m’HQ_m_ statistics (0.77284; *p* < 0.001))

Based on the β-diversity analysis, bacterial communities were strongly clustered according to the origin of the tissue distinguishing the gut bacterial profile with that from the reproductive organs (Fig. 7a) (PERMANOVA; *p* < 0.001). This factor explained 86.7% of the total variance. The bacterial communities seem to be affected statistically by the developmental stage and age of the insects with the 1-day, 15-day, and the larval bacterial profile clustering separately (Fig. 7b) (PERMANOVA, *p* < 0.001). CAP ordinations were supported by significant trace_Q_m’HQ_m_ statistics (0.77284; *p* < 0.001). Interestingly, the origin of the laboratory colonies does not seem to be a critical factor for the shaping of the bacterial profile in the gastrointestinal tract and the gonads of the insects (PERMANOVA; *p* < 0.074) (see Additional file 4). Similarly, no differences were observed between the bacterial associations between males and females (PERMANOVA; *p* < 0.062).

## Discussion

In the present work, we investigated the bacterial profile of three different *Gpg* laboratory colonies (BKF, SEN, BKF-SEN) using a next-generation sequencing approach. *Wigglesworthia* and *Sodalis* were the most dominant taxa in all examined samples. Relative abundance was influenced by tissue type with *Wigglesworthia* being mainly dominant in adult gut tissues while gonads were characterized by the dominance of *Sodalis*. In larval gut samples, a balanced co-existence between *Wigglesworthia* and *Sodalis* was observed. Previously, Rio and colleagues showed that the two beneficial partners *Wigglesworthia* and *Sodalis* in a laboratory colony of *Glossina morsitans morsitans* exhibited well-regulated density profiles through host development and diverse disruptive events (such as host immune challenge and environmental perturbations), while *Wolbachia* density displayed wide variability through the development, highlighting the importance of co-adaptive processes, the age of the association and the functional arsenal of symbionts [26].

In addition to the two dominant species (*Wigglesworthia and Sodalis*), several other genera were detected including *Acinetobacter, Bacillus, Brevundimonas, Enterococcus, Exiguobacterium, Flavobacterium, Klebsiella, Providencia, Pseudomonas* and *Staphylococcus*, as reported in previous studies [10, 11, 21, 22, 24, 25]. Also, *Wolbachia* was detected in the majority of the *Gpg* samples studied but in very low titers, while *Spiroplasma* was absent, in accordance with previous studies [10, 17]. However, *Serratia* was not detected in any examined *Gpg* samples, although this bacterium had been isolated from the midgut of insectary *Glossina palpalis gambiensis* flies originated from individuals that had been field-collected in Burkina Faso [23].

Our data indicated that the origin of the three examined *Gpg* laboratory colonies did not affect the composition of the symbiotic profile. So, the poor performance of tsetse flies observed in the past in the BKF-SEN colony may be due to genetic factors and not to the associated microbiota [9], although a comparison with the gut microbiota of natural populations has not been performed. Overall, the three examined *Gpg* laboratory colonies exhibited similar species richness and diversity indices. Furthermore, in all bacterial taxa examined at phylum and the 24 most dominant taxa at genus (data not shown), no statistically significant divergence in relative abundance was observed in relation to the origin of the three laboratory colonies. Both the maternally transmitted obligate and commensal dominant symbionts *Wigglesworthia* and *Sodalis*, as well as the environmentally acquired bacteria are not affected by the origin of the populations and present similar dynamics (qualitatively and semi-quantitatively). This indicated a high degree of homogeneity for the three *Gpg* laboratory colonies in relation to their bacterial profile and the quantitative distribution of the bacteria that coexisted with them once they were established under mass-rearing conditions. In this respect, it is worth noting that the long domestication period for the BKF colony (> 35 years under laboratory conditions) did not have any impact on the bacterial profile as compared with the recently established SEN and BKF-SEN colonies. This might be due to the nature of the feeding type of these flies which were largely on sterile blood under laboratory conditions.

In *Drosophila melanogaster* different population backgrounds (genotype and origin) exhibited significant differences in the established host microbiome. This was regardless the long-term detrimental effect of diet [27]. Different Chinese natural populations of *Bactrocera dorsalis* displayed different bacterial diversity [28]. This bacterial diversity was dependent on the time of the pest invasion in the region, with the wild flies harboring significant higher bacterial richness than the laboratory-reared flies [28]. Similarly, a previous study revealed that different living environments and diets affect the bacterial composition of *Bactrocera dorsalis* when field and laboratory populations were compared [29]. Additionally, a recent study suggested that diet is the main determinant for gut bacterial profile in two wood-feeding beetles, the *Monochamus alternatus* and *Psacothea hilaris*, [30].

Our results indicated that the distribution of bacteria in *Gpg* laboratory colonies was based on the type of tissue examined. In particular, the gut samples displayed statistically significant lower species-richness than the gonads, which can be attributed to the sterile blood meal provided to the *Gpg* laboratory colonies which was considered as a limited bacterial source. A large-scale study showed that omnivorous insects harbor significantly higher level of bacterial diversity than stenophagous (carnivorous and herbivorous) insects [31].

Our study indicated that age and gender did not affect significantly neither the species richness/diversity of the *Gpg* laboratory colonies nor the relative abundance of the most dominant taxa in all analyzed samples. This is probably due to the standard rearing conditions applied to all laboratory colonies examined regardless of the developmental stage and it can be attributed to the unique lifestyle of tsetse fly [10, 32]. Only, in teneral *Gmm* flies a significant increase of *Wigglesworthia* and *Wolbachia* was observed while the dynamics of the two symbionts remained stable through the other developmental stages [26]. This could be due to the immature immunity system of *Gmm* in teneral stage which allows bacterial proliferation [26]. This advocates that the transition to a life stage independent of the maternal environment is pivotal for tsetse’s bacterial dynamics. The larval gut samples showed statistically significant higher species-richness and diversity from the adult gut samples. Recently, in the herbivore *Spodoptera littoralis* a similar significant reduction in bacterial diversity was observed during the development from egg to pupa, highlighting the effect the developmental stage has over the gut bacterial flora [32]. Interestingly, in honey bee multiple factors including ontogenetic stage, age and geographic location have been shown to affect the gut microbiota [33].

## Conclusions

Although the bacterial diversity of natural populations and laboratory colonies of tsetse fly seem to be limited [10, 21], additional research is required to reveal the role of the environmentally acquired bacteria in the physiology of their host. Our data indicated that regardless of the origin of the laboratory colonies, once they enter common rearing practices and kept on the same diet, gut and gonadal microbiota is relatively stable. This can be extremely useful in future attempts to develop and maintain suitable laboratory colonies that will be enriched with desired bacterial strains or free from harmful bacteria. A similar approach has been applied successfully in *Anopheles gambiae*, which is the main vector of human malaria (*Plasmodium* sp.) by the exploitation of isolations of the genera *Enterobacter* and *Serratia* with anti-Plasmodium activity [34–37]. However, it should be noted that the present study has focused on only three laboratory colonies of a single species and should be expanded in order to unravel the role of microbiota in tsetse flies’ biology and vector competence towards the potential harnessing of the naturally associated bacterial richness in vector and disease control.

## Methods

### Sample collection-dissection-DNA extraction

*Gpg* laboratory colonies from IPCL were used (see Additional file 5): (a) BKF, (b) SEN, and (c) BKF-SEN. Tissues from the laboratory reared tsetse flies were freshly dissected in PBS under sterile conditions and DNA extraction was performed as described previously [38]. In brief, tsetse flies (both adult and larvae) were surface-sterilized prior to dissection by dipping once in 70% ethanol and once in 1× sterile PBS. DNA was extracted using the Qiagen DNeasy kit (Qiagen, Valencia, CA). DNA samples were kept at -20 °C. Tissue collections including guts and reproductive organs (testes and ovaries) and were made during July and August 2013. Guts were collected from third instar larvae and both teneral and 15 day-old males and females. Reproductive tissues were collected from teneral and 15 day-old flies (males and females). Samples were pooled from five individuals and three biological replicates were collected per sample. The quantity and quality of the DNA was measured using NanoDrop 1000 (Thermo Scientific).

### Multiplex Illumina MiSeq sequencing, data, and statistical analysis

Fusion primers U341F (5’-CCTACGGGRSGCAGCAG-3′), and 805R (5’-GACTACCAGGGTATCTAAT-3′) were used to amplify the V3-V4 of the 16S *rRNA* gene [39]. 805R reverse primers contained a unique Golay barcode specific to each sample for read de-multiplexing. PCR reactions were carried out in a volume of 25 μl containing 1 μl of template DNA, 12.5 μl NEBNext 2× High-Fidelity Master Mix (New England Biolabs, UK), 1 μl of each primer at 3 mM concentration and 9.5 μl PCR-clean H_2_O. The cycling conditions used were 30 s at 98 °C, 25 cycles of 98 °C for 10 s, 58 °C for 30 s and 72 °C for 30 s, followed by a final extension of 5 min at 72 °C, resulting in an amplicon of approximately 465 bp For the above PCR reactions, the appropriate negative (no DNA) and positive controls were included. Amplicons were cleaned using Ampure XP beads (Agencourt, UK), and re-suspended in 30 μl PCR-clean H_2_O. Products were quantified using the Qubit dsDNA High-Sensitivity assay (Life Technologies, UK), and an Agilent Bioanalyzer High-Sensitivity DNA chip (Agilent, UK). Samples were pooled at equimolar concentrations (300 ng per sample) for size-selection by Pippin-Prep (Sage Science, UK), where a size range of 350–450 bp was extracted. Size-selected fragments underwent the same clean-up and quantification steps as above prior to sequencing. Sequencing was performed at IMGM Laboratories GmbH on an Illumina MiSeq platform using 300 bp paired-end read chemistry (Illumina, USA).

Raw sequencing reads were de-multiplexed, converted to FASTQ, and the Illumina adapters were trimmed using Illumina standard algorithms. Paired-end reads were assembled, trimmed by length and further corrected for error and quality using the usearch -fastq_mergepairs option. All subsequent analyses were conducted in usearch version 10 [40]. Briefly, the quality of the assembled sequences was further improved using the -fastq_filter, followed by finding unique read sequences and abundances by using the -fastx_uniques option. Sequences were clustered into OTUs using the -cluster_otus command [40]. Chimeras were removed using the -unoise3 option [41]. Taxonomy was assigned to using the sintax option against the SILVA 128 release database [42, 43].

Beta-diversity analysis was calculated using weighted Unifrac distances, and PCoA analyses were performed on the resulting distance matrix. These calculations and those for alpha diversity were performed in QIIME version 1.9.1 and PRIMER6+. PERMANOVA tests were employed to detect statistical differences. Overall similarities in bacterial community structures were shown using the unconstrained ordination technique of Principal Coordinate Analysis (PCA). Differences in community structure were viewed using the constrained ordination technique of Canonical Analysis of Principal coordinates (CAP), tested using the CAP classification success rate and CAP trace_Q_m’HQ_m_ statistics, and were performed with 9999 permutations within PRIMER6 + .

## Additional files

Additional file 1: Richness and diversity estimation of the 16S *rRNA* gene libraries from the amplicon sequence analysis of the three *Gpg* laboratory colonies. (DOCX 24 kb)

Additional file 2: Relative abundance of *Wigglesworthia*, *Sodalis* and *Streptococcus* for the three laboratory colonies examined over time (15 day-old adults, 1 day-old adults and larva). (JPG 21 kb)

Additional file 3: Relative abundance of *Wigglesworthia*, *Sodalis* and *Streptococcus* for the three laboratory colonies examined in relation to the gender and developmental stage (Female, Larva and Male). (JPG 15 kb)

Additional file 4: Principal Coordinates Analysis (PCoA) of bacterial communities based on relative abundances of OTUs with ordinations from *Gpg* laboratory colonies of the three laboratory colonies examined. Variance explained by each PCoA axis is given in parentheses. (JPG 28 kb)

Additional file 5: Collection protocol and sample description. (DOCX 16 kb)

## Abbreviations

AAT: Animal African Trypanosomiasis
AW-IPM: Area-Wide Integrated Pest Management
BKF: Burkina Faso
BKF-SEN: SEN males were outcrossed to BKF females to produce a tsetse line
CAP: Canonical Analysis of Principal coordinates
*Gff*: *Glossina fuscipes fuscipes*
*Gmed*: *Glossina medicorum*
*Gmm*: *Glossina morsitans morsitans*
*Gms*: *Glossina morsitans submorsitans*
*Gpal*: *Glossina pallidipes*
*Gpg*: *Glossina palpalis gambiensis*
*Gpp*: *Glossina palpalis palpalis*
*Gt*: *Glossina tachinoides*
IPCL: Insect Pest Control Laboratory
OTUs: Operational Taxonomic Units
PBS: Phosphate Buffer Saline
PCoA: Principal Coordinates Analysis
PCR: Polymerase Chain Reaction
SEN: New colony from flies collected in the Niayes
SIT: Sterile Insect Technique
ΗΑΤ: Human African Trypanosomiasis
