# Supplementary material for: Different laboratory populations similar bacterial profile? The case of Glossina palpalis gambiensis
Source: BMC Microbiol. 2018 Nov 23;18(Suppl 1):148. doi: 10.1186/s12866-018-1290-9 (PMC6251098; doi:10.1186/s12866-018-1290-9)
Supplement: Supplementary file 1 — Richness and diversity estimation of the 16S rRNA gene libraries from the amplicon sequence analysis of the three Gpg laboratory colonies. (DOCX 24 kb) [file 12866_2018_1290_MOESM1_ESM.docx]

**Additional Files**

**Additional File 1:** Richness and diversity estimation of the 16S *rRNA* gene libraries from the amplicon sequence analysis of the three *Gpg* laboratory colonies.

| **Sample** | | | | | | **Number of OTUs** | **Species richness indices** | **Species diversity indices** | |
| --- | --- | --- | --- | --- | --- | --- | --- | --- | --- |
| **Barcode name** | **Lab colony** | **Tissue** | **Age** | **Stage /Sex** | **Origin** |  | **Chao1** | **Shannon** | **Simpson** |
| BGM2 | BKF | Gut | 1d | MALE | Burkina | 25.17±1.83 | 36.22±2.70 | 0.84±0.04 | 0.37±0.020 |
| BTM2 | BKF | Testes | 1d | MALE | Burkina | 55.43±0.42 | 62.61±1.24 | 1.08±0.03 | 0.28±0.013 |
| BGF3 | BKF | Gut | 1d | FEMALE | Burkina | 24.03±1.49 | 34.13±2.37 | 0.65±0.04 | 0.26±0.021 |
| BOF3 | BKF | Ovaries | 1d | FEMALE | Burkina | 60.20±1.27 | 73.25±2.13 | 1.10±0.03 | 0.27±0.008 |
| BGM8 | BKF | Gut | 15d | MALE | Burkina | 11.07±0.55 | 24.29±4.11 | 0.75±0.02 | 0.34±0.015 |
| BTM8 | BKF | Testes | 15d | MALE | Burkina | 67.13±1.38 | 76.58±1.85 | 0.94±0.03 | 0.22±0.008 |
| BGF9 | BKF | Gut | 15d | FEMALE | Burkina | 11.93±0.87 | 22.76±2.04 | 0.76±0.01 | 0.32±0.009 |
| BOF9 | BKF | Ovaries | 15d | FEMALE | Burkina | 67.70±0.85 | 81.06±1.78 | 0.87±0.03 | 0.22±0.009 |
| IGM2 | BKF-SEN | Gut | 1d | MALE | Intro | 20.83±1.60 | 30.48±2.91 | 0.57±0.04 | 0.22±0.019 |
| ITM2 | BKF-SEN | Testes | 1d | MALE | Intro | 80.83±1.69 | 86.58±1.74 | 1.72±0.02 | 0.42±0.021 |
| IGF3 | BKF-SEN | Gut | 1d | FEMALE | Intro | 12.73±1.04 | 23.05±2.46 | 0.24±0.01 | 0.07±0.003 |
| IOF3 | BKF-SEN | Ovaries | 1d | FEMALE | Intro | 80.67±3.39 | 87.96±3.61 | 2.14±0.11 | 0.58±0.019 |
| IGM8 | BKF-SEN | Gut | 15d | MALE | Intro | 13.07±0.49 | 31.93±4.59 | 0.85±0.02 | 0.39±0.012 |
| ITM8 | BKF-SEN | Testes | 15d | MALE | Intro | 82.63±0.69 | 108.46±2.81 | 0.92±0.03 | 0.23±0.012 |
| IGF9 | BKF-SEN | Gut | 15d | FEMALE | Intro | 13.33±0.47 | 33.36±4.38 | 0.52±0.01 | 0.20±0.003 |
| IOF9 | BKF-SEN | Ovaries | 15d | FEMALE | Intro | 77.93±0.79 | 100.86±2.23 | 1.19±0.06 | 0.37±0.025 |
| SGM2 | SEN | Gut | 1d | MALE | Senegal | 21.10±1.09 | 36.95±1.86 | 0.63±0.04 | 0.26±0.023 |
| STM2 | SEN | Testes | 1d | MALE | Senegal | 77.80±1.18 | 86.68±1.71 | 1.45±0.02 | 0.33±0.007 |
| SGF3 | SEN | Gut | 1d | FEMALE | Senegal | 12.17±0.42 | 22.27±1.66 | 0.37±0.01 | 0.13±0.002 |
| SOF3 | SEN | Ovaries | 1d | FEMALE | Senegal | 67.03±0.48 | 79.15±1.21 | 1.22±0.04 | 0.35±0.022 |
| SGM8 | SEN | Gut | 15d | MALE | Senegal | 13.30±0.27 | 21.58±1.45 | 0.82±0.002 | 0.37±0.001 |
| STM8 | SEN | Testes | 15d | MALE | Senegal | 72.97±0.91 | 89.73±2.26 | 0.73±0.02 | 0.15±0.007 |
| SGF9 | SEN | Gut | 15d | FEMALE | Senegal | 11.27±0.62 | 24.37±2.20 | 0.57±0.01 | 0.23±0.006 |
| SOF9 | SEN | Ovaries | 15d | FEMALE | Senegal | 79.37±1.18 | 101.49±3.23 | 1.46±0.03 | 0.52±0.005 |
| BGL1 | SEN | Gut | Larva | LARVA | Burkina | 32.00±1.95 | 50.19±3.70 | 1.03±0.01 | 0.48±0.004 |
| IGL1 | SEN | Gut | Larva | LARVA | Intro | 41.03±1.66 | 65.77±3.37 | 1.04±0.02 | 0.45±0.007 |
| SGL1 | SEN | Gut | Larva | LARVA | Senegal | 43.33±1.32 | 57.38±1.59 | 1.09±0.01 | 0.49±0.002 |
