# Supplementary figures and images for: Different laboratory populations similar bacterial profile? The case of Glossina palpalis gambiensis
Source: BMC Microbiol. 2018 Nov 23;18(Suppl 1):148. doi: 10.1186/s12866-018-1290-9 (PMC6251098; doi:10.1186/s12866-018-1290-9)

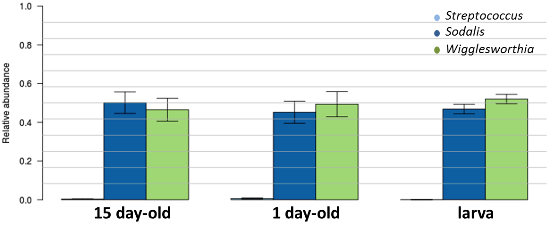

Supplement: Supplementary file 2 — Relative abundance of Wigglesworthia, Sodalis and Streptococcus for the three laboratory colonies examined over time (15 day-old adults, 1 day-old adults and larva). (JPG 21 kb) [file 12866_2018_1290_MOESM2_ESM.jpg]

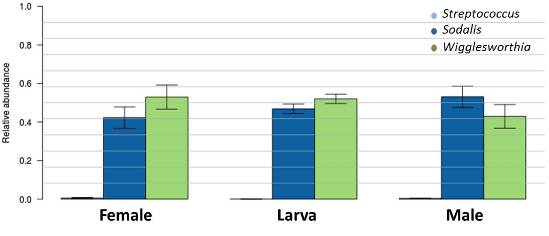

Supplement: Supplementary file 3 — Relative abundance of Wigglesworthia, Sodalis and Streptococcus for the three laboratory colonies examined in relation to the gender and developmental stage (Female, Larva and Male). (JPG 15 kb) [file 12866_2018_1290_MOESM3_ESM.jpg]

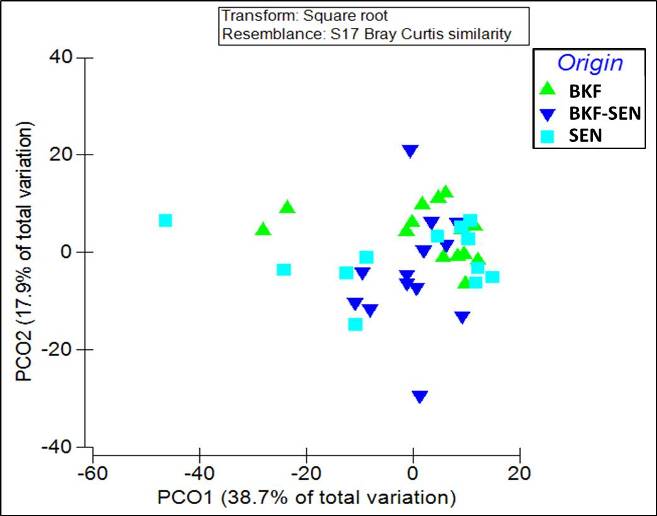

Supplement: Supplementary file 4 — Principal Coordinates Analysis (PCoA) of bacterial communities based on relative abundances of OTUs with ordinations from Gpg laboratory colonies of the three laboratory colonies examined. Variance explained by each PCoA axis is given in parentheses. (JPG 28 kb) [file 12866_2018_1290_MOESM4_ESM.jpg]
