# Supplementary material for: Different laboratory populations similar bacterial profile? The case of Glossina palpalis gambiensis
Source: BMC Microbiol. 2018 Nov 23;18(Suppl 1):148. doi: 10.1186/s12866-018-1290-9 (PMC6251098; doi:10.1186/s12866-018-1290-9)
Supplement: Supplementary file 5 — Collection protocol and sample description. (DOCX 16 kb) [file 12866_2018_1290_MOESM5_ESM.docx]

**Additional File 5**: Collection protocol and sample description.

| **Species** | **Colony** | **Tissue** | **Larvae** | **Adults** | | | |
| --- | --- | --- | --- | --- | --- | --- | --- |
|  |  |  |  | **Males** | | **Females** | |
|  |  |  |  | **1-day** | **15-day** | **1-day** | **15-day** |
| *G. p. gambiensis* | BFK | Gut | 3x5 | 3x5 | 3x5 | 3x5 | 3x5 |
|  |  | Testis |  | 3x5 | 3x5 |  |  |
|  |  | Ovaries |  |  |  | 3x5 | 3x5 |
|  | SEN | Gut | 3x5 | 3x5 | 3x5 | 3x5 | 3x5 |
|  |  | Testis |  | 3x5 | 3x5 |  |  |
|  |  | Ovaries |  |  |  | 3x5 | 3x5 |
|  | BFK-SEN | Gut | 3x5 | 3x5 | 3x5 | 3x5 | 3x5 |
|  |  | Testis |  | 3x5 | 3x5 |  |  |
|  |  | Ovaries |  |  |  | 3x5 | 3x5 |
